# Unrecognised depression among older people: a cross-sectional study from Norwegian general practice

**DOI:** 10.3399/BJGPO.2022.0135

**Published:** 2023-02-22

**Authors:** Lars Christian Kvalbein-Olsen, Eivind Aakhus, Ole R Haavet, Erik L Werner

**Affiliations:** 1 Department of General Practice, Faculty of Medicine, University of Oslo, Oslo, Norway; 2 Department of Old Age Psychiatry, Innlandet Hospital Trust, Brumunddal, Norway

**Keywords:** depression, care of older people, aged, epidemiology, general practice, primary healthcare

## Abstract

**Background:**

Depression is common in old age and is associated with disability, increased mortality, and impairment from physical diseases.

**Aim:**

To estimate the prevalence of depression among older patients in Norwegian general practice, to evaluate the extent they talk about it during their consultation, whether it was previously known or suspected by their GP, and how frequently patients with depression visit their GP.

**Design & setting:**

Cross-sectional study among patients and GPs at 18 primary care clinics in the south of Norway.

**Method:**

Patients aged ≥65 years who visited their GP were asked to complete the Patient Health Questionnaire-9 (PHQ-9). The GPs reported what kind of issues the patient presented at the consultation, if a current depression was known, and the consultation frequency.

**Results:**

Forty-four (11.4%) of 383 patients reported moderate or severe depressive symptoms (PHQ-9 ≥10). Among the cases with data from both patient and GP (*n* = 369), 38 patients (10.3%) reported moderately depressive symptoms. Of these, only 12 (31.6%) mentioned psychological problems to their GP during their consultation; 12 (31.6%) with previous depression were neither known to the GP nor suspected of currently having depression; and 67.6% of them visited their GP ≥5 times a year.

**Conclusion:**

Older patients tend to speak little of their depression to the GP. Almost one in three older patients with moderate depressive symptoms were unrecognised by their GP. Older patients who frequently visit the GP should be suspected of potentially having mental health problems.

## How this fits in

Depression is common in older patients in general practice, but often it seems to be masked by other symptoms or neglected by both patients and GPs. This cross-sectional study shows that mental health problems were addressed in only 9.2%of the consultations, and, even among the patients bearing moderately depressive symptoms, only 31.6% addressed this in the consultation. As this study also found that almost one in three moderately depressed older patients was unrecognised by their GP, there should be a change in practice to address the lack of recognition.

## Introduction

Depression is common in older patients all over the world,^1^ and leads to a greater reduction in patients' overall health status than other chronic health conditions.^2^ Contrary to young people, depression in older people presents with less affective symptoms, and is more likely to be seen as cognitive changes, loss of interest,^3^ anxiety,^4^ and somatic symptoms.^3,4^ The symptoms of depression in older people often seem to be masked by unexplained physical ailments — such as fatigue, diffuse pain, back pain, headaches, and chest pain — and diagnostics, and they can be somewhat more complicated than in the general adult population.^5^

The prevalence of depression among older patients is high in most studies, but heterogeneity among the studies makes comparisons challenging.^1^ Thus, reported prevalences show considerable variation as wide as 1%–32%,^1,6–9^ owing to different methods and populations. Some studies are based on clinical interviews fulfilling diagnostic criteria, while others use various psychometric scales to detect the depressive symptoms of the patient.

The largest risk factors for depression among middle-aged and older persons were found to be self-perceived social isolation and self-reported poor health, as well as difficulties with instrumental activities in daily life (men) and increased family burden (women).^10^ Cole and Dendukuri found the risk factors for depression among older people to be disability, new medical illness, poor health status, previous depression, poor self-perceived health, and bereavement.^11^

There are two widely used diagnostic systems for depression, *International Statistical Classification of Diseases and Related Health Problems* (ICD-10)^12^ and the *Diagnostic and Statistical Manual of Mental Disorders, Fifth Edition* (DSM-5),^13^ with minor variations. ICD-10 is the official international classification in psychiatry, but the DSM-5 is used more often in the US and for treatment research purposes. The PHQ-9^14^ is a widely used self-administered screening tool for depression in general practice, which is based on all of the nine main DSM-5 criteria for depression and shows good accuracy.^15^

GP consultations often cover multiple problems,^16–18^ and, for the general population, mental health issues are presented in one-quarter of the consultations.^17,18^ However, owing to time constraints at the consultation and the complex needs of older people, physical health complaints are often prioritised over mental health.^19^

In general practice, depression in older patients is often undetected and/or untreated.^20^ Reasons for this may be stigma associated with depression,^21,22^ lack of recognition, and false beliefs that the experience of depressive symptoms are part of natural ageing.^23,24^ The consequences of untreated depression in older people can be poor quality of life, worsening of chronic diseases, increased mortality, and suicide.^25,26^

To date, there has been little research on these 'unrecognised depressions' in older patients.^27^ Some studies on the general adult population have found that the GPs correctly identified only almost half of the depressed adult patients.^28,29^ However, there is little research on whether older patients reveal their current depressive symptoms at the consultation, or whether GPs are aware of patients' depression if these symptoms are not specifically mentioned by the patient.

Therefore, this study aimed to explore the frequency of depression among patients aged ≥65 years, and to what extent older patients with depression address their current psychological issues at their GP consultation. The study also explored whether GPs were familiar with older patients having depression regardless of whether it was brought into the consultation. Finally, the study aimed to investigate how frequently the patients with depression visit their GP.

## Method

### Design and procedure

In 2019, GPs in southern Norway were invited to take part in this study, through the professional network, direct requests, and by social media. All data were collected from November 2019–July 2021.

During a 2-month period, the GPs were asked to invite every consulting patient aged ≥65 years to respond to a questionnaire, after the consultation. The GPs themselves were also asked to respond to a short form about their knowledge about the patient and the topics discussed at the consultation. It was a step-wise inclusion of GPs during the whole period, and it could be prolonged if more responders were needed.

Inclusion criteria were patients aged ≥65 years, home-dwelling and visiting the GP for any reason. Exclusion criteria were lack of consent from the patient. If needed (for example, any reading and/or writing difficulties) the patient could be assisted in completing the form.

Owing to the low number of responders during this first data collection (sample 1), eight practices were asked to continue data collection by delivering questionnaires directly from the secretary at the reception. It was done by informing all the receptionists to offer the same questionnaire to all visiting patients aged ≥65 years, but without the GP part (sample 2). It was thought this could also contribute to informing the authors of a potential selection bias of patients with depression from the GPs in sample 1.

### Measures

In addition to demographics and general health issues, the patient questionnaire consisted of three validated questionnaires.

The PHQ-9 was used for depressive symptoms, and consists of nine items, which are scored 0–3 points on a Likert scale, with a ≥10 point cut-off indicating moderate depression.^14^ It has been translated and validated into many languages, and it is also used for older patients.^30^ Recently reference data for an adult Norwegian population have been published.^31,32^

The General Anxiety Disorder 7-item scale (GAD-7)^33^ was used for anxiety symptoms. It is also validated in Norwegian^34^ and for use in older people.^35^ The questionnaire consists of seven items, which are scored 0–3 points on a Likert scale, with a ≥10 point cutoff indicating a general anxiety disorder.^33^

For somatic burden purposes, subjective health complaints (SHC) was used, which is a 29-item scale measuring subjective somatic and psychological complaints during the past 30 days without reference to specific diagnosed categories.^36^ It is also validated in older patients.^37^ A scale of 0 (no complaints) to 3 (severe complaints) is used, giving a total score from 0 (excellent) to 87 (very poor).^36,38^ No defined cut-off is established.

The GPs were asked to record topics that were addressed during the consultation. Since there is no standardised short categorisation or questionnaire for this, an adapted version of Bjørland *et al*’s categorisation was used,^18^ with the following categories: (1) pain condition; (2) somatic problem; (3) mental problem; (4) social causes; (5) sleep problem; (6) routine control; (7) administrative matter; and (8) other. The GP was also asked whether they were familiar with any depression the patient had experienced in the past 12 months, or whether they thought the patient might have a current depressive disorder, regardless of whether this was discussed during the consultation. The GPs were asked to register the number of visits from the patient during the past 12 months (categorised as 0–1, 2–4, and ≥5).

### Analysis

All data were calculated and analysed statistically by SPSS (version 28), using simple descriptive analysis. For continuous variables, mean and standard deviation (SD) were calculated. Frequencies and percentages were calculated for categorical variables.

## Results

**Figure 1.**
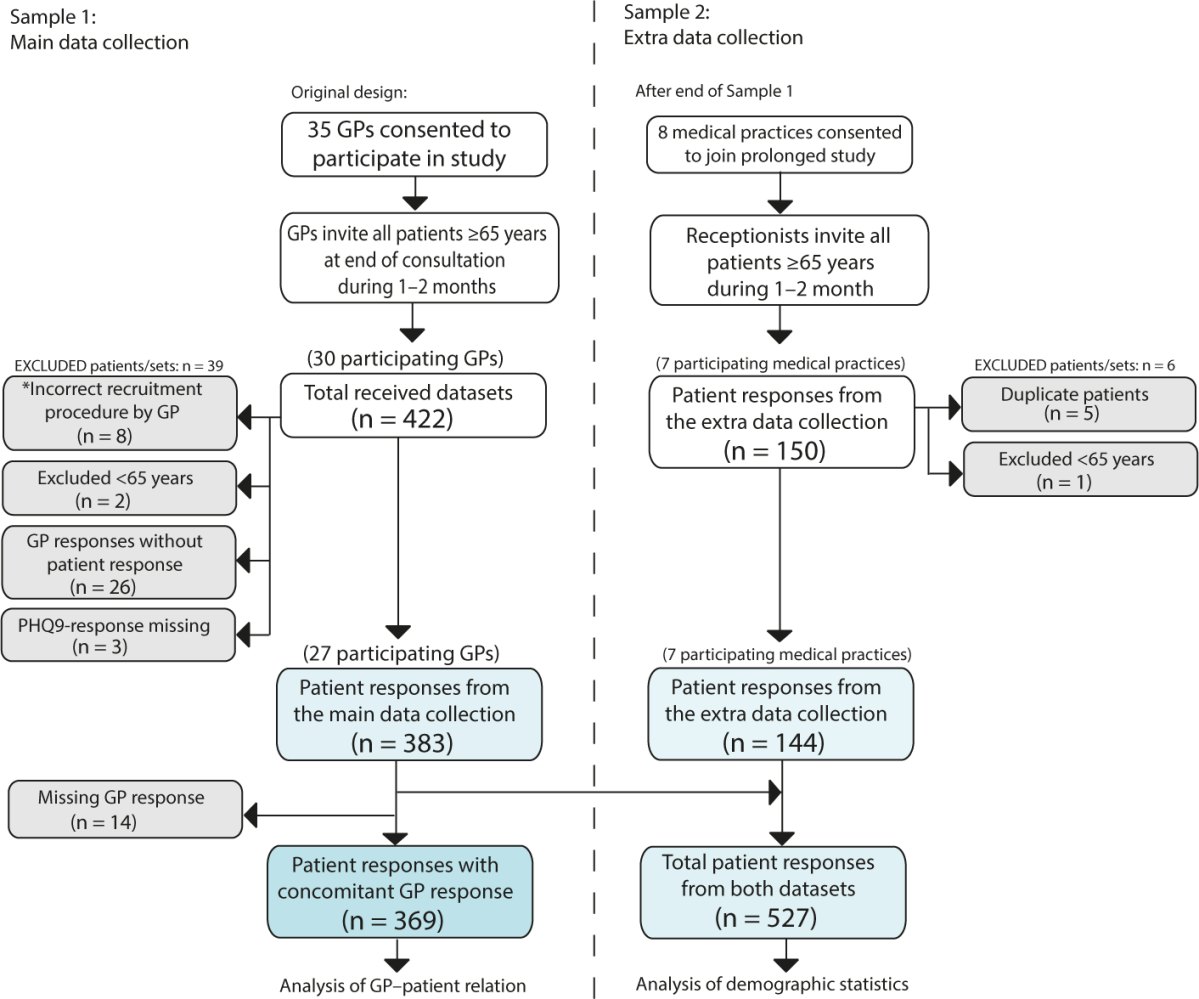
Flow chart of recruitment process and data collection. PHQ-9 = Patient Health Questionnaire-9. ^*^Three GPs misunderstood the recruitment procedure, selectively recruiting only patients with depression (instead of every patient aged ≥65 years).

A total of 383 patient responses were received in sample 1, of which 369 cases were complete data from both GPs and patients (Figure 1). In addition, 144 patient responses were received in sample 2. Mean age of the patients overall was 74.5±6.6 years and 56.5% were women. The mean age of the 27 GPs was 42.5±6.8 years, and consisted of 51.9%women. Seventy per cent were specialists in general practice and they included approximately 14.2 patients each (range 2–42).

### Main findings

It was found that 11.4% of the patients reported moderate depressive symptoms or worse (PHQ-9 ≥10) (*Table 1*). There were no significant differences between the two samples (*Table 2*).

**Table 1.** Patient characteristics, and prevalence of moderate-to-severe depressive symptoms

Group	Part of total (*N*’)		Prevalence of PHQ-9≥10 in group		Numbers answered on item
Patient answers	***N***	**% (*N/N*’**)	***n***	**% (*n/N***)	
**Total patient samples:**	527		60	11.4%	
Part 1 total:	383		44	11.5%	
Part 1 complete sets	369		43	11.7%	
Part 1 GP parts missing	14		1	N/A	
Part 2 :	144		16	11.1%	
**Sex**					
Female	298	56.5%	42	14.1% (of 298)	527
Male	229	43.5%	18	7.9% (of 229)	527
**Age, years**					
65–69	140	26.6%	18	12.9% (of 140)	527
70–74	144	27.3%	13	9.0% (of 144)	527
75–79	125	23.7%	14	11.2% (of 125)	527
80–84	75	14.2%	9	12.0% (of 75)	527
≥85	43	8.2%	6	14.0% (of 43)	527
**Marital status**					
Married or cohabitant	337	63.9%	36	10.7% (of 337)	520
Widowed	106	20.1%	17	16.0% (of 106)	520
Divorced	49	9.3%	4	8.9% (of 49)	520
None	33	6.3%	3	9.1% (of 33)	520
**Various**					
Live near (grand)children	401	78.0%	54	13.5% (of 401)	514
Foreign native language	21	4.1%	6	28.6% (of 21)	515
Smokes	53	10.3%	16	30.2% (of 53)	513
Hypnotics	132	25.6%	25	18.9% (of 132)	516
Anxiolytics	54	10.5%	19	35.2% (of 54)	516
Home-based nursing service	33	6.4%	12	36.4% (of 33)	516
**Patient reported symptoms**					
SHC ≥20 (9 = median)	89	16.9%	59	23.2% (of 254)	526
Probable general anxiety disorder:					
GAD-7≥10	40	7.6%	29	72.5% (of 40)	527
N/A					
**GPs answers (total *n* = 369):**					
**GP stated consultation topics**	***N***	***% (N/N’)***	***n***	***% (n/N)***	***(N’)***
Routine control	134	36.6%	8	6.0% (of 134)	369
Somatic problem	214	58.0%	24	11.2% (of 214)	369
Pain problem	61	16.5%	10	16.4% (of 61)	369
Mental health problem	34	***9.2%***	15	44.1% (of 34)	369
Social or relational problem	18	4.9%	7	38.9% (of 18)	369
Sleep problem	18	4.9%	5	27.8% (of 18)	369
**Consultation frequency**					
Frequent patient (≥ x5/year)	163	46.4%	28	17.2% (of 163)	351
Occasionally (2–4 x/year)	140	39.9%	7	5.0% (of 140)	351
Seldom (0–1 x/year)	48	13.7%	4	8.3% (of 48)	351

GAD-7 = General Anxiety Disorder 7-item scale. PHQ-9 = Patient Health Questionnaire-9. SHC = subjective health complaints.

Missing data: 7 patients did not answer demographic questions, but did complete the PHQ-9 and had completed GP forms. These were, therefore, included in analysis.

**Table 2.** Sample variation between the two different recruitment methods

	Sample 1+2:		Sample 1:		Sample 2:	
	*n*	*%*	*n*	*%*	*n*	%
**Total responders without GP part**	527		383		144	
**Sex**						
Female	298	56.5%	230	60.1%	68	47.2%
Male	229	43.5%	153	39.9%	76	52.8%
**Age, years**						
65–69	140	26.6%	103	26.9%	37	25.7%
70–74	144	27.3%	107	27.9%	37	25.7%
75–79	125	23.7%	83	21.7%	42	29.2%
80–84	75	14.2%	59	15.4%	16	11.1%
≥85	43	8.2%	31	8.1%	12	8.3%
**Depressive (PHQ-9) level**						
None (<5)	341	64.7%	248	64.8%	93	64.6%
Mild (5–9)	126	23.9%	91	23.8%	35	24.3%
Moderate (10–19)	53	10.1%	39	10.2%	14	9.7%
Severe (20–29)	7	1.3%	5	1.3%	2	1.4%
						
**Mean values**	**Mean**	**± SD**	**Mean**	**± SD**	**Mean**	**± SD**
Mean age	74.50	±6.6 years	74.54	±6.6 years	74.48	±6.7 years
Mean SHC score	11.15	±8.95	11.26	±9.07	10.92	±8.65
Mean PHQ-9 score	4.36	±4.44	4.30	±4.39	4.55	±4.59
Mean GAD-7 score	2.87	±3.64	2.71	±3.37	3.28	±4.27

GAD-7 = General Anxiety Disorder 7-item scale. PHQ-9 = Patient Health Questionnaire-9. SHC = subjective health complaints.

Mental health issues were addressed in 9.2% of the consultations (*Supplementary Table S1*).

**Figure 2.**
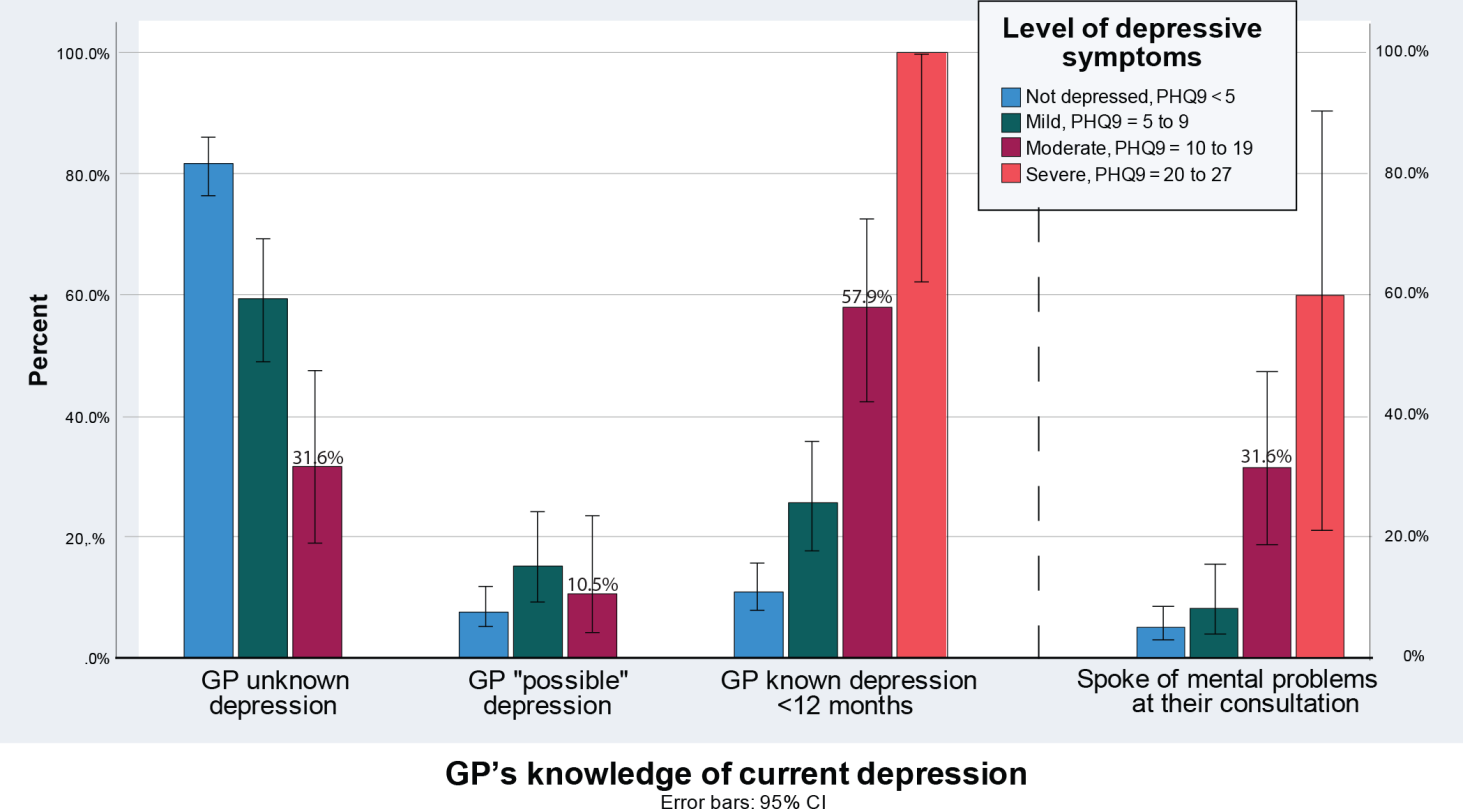
GP’s knowledge of a current depression, related to Patient Health Questionnaire-9 score.

Among the patients with a PHQ-9 score from 10–19, 10.3% reported moderate depressive symptoms, and 68.4% did not talk about mental health issues during their consultation (*Supplementary Table S1*). In 57.9% of the cases with moderate depression, this was previously recognised by the GPs (*Supplementary Table S1 and Figure 2*). In addition, the GPs suspected 10.5% of them to possibly have depression even if they did not talk about it (*Supplementary Table S1 and Figure 2*). Still, 31.6% of patients with a probable depression were neither known to have a current depression, nor was this suspected or addressed by the GP (*Supplementary Table S1 and Figure 2*).

**Figure 3.**
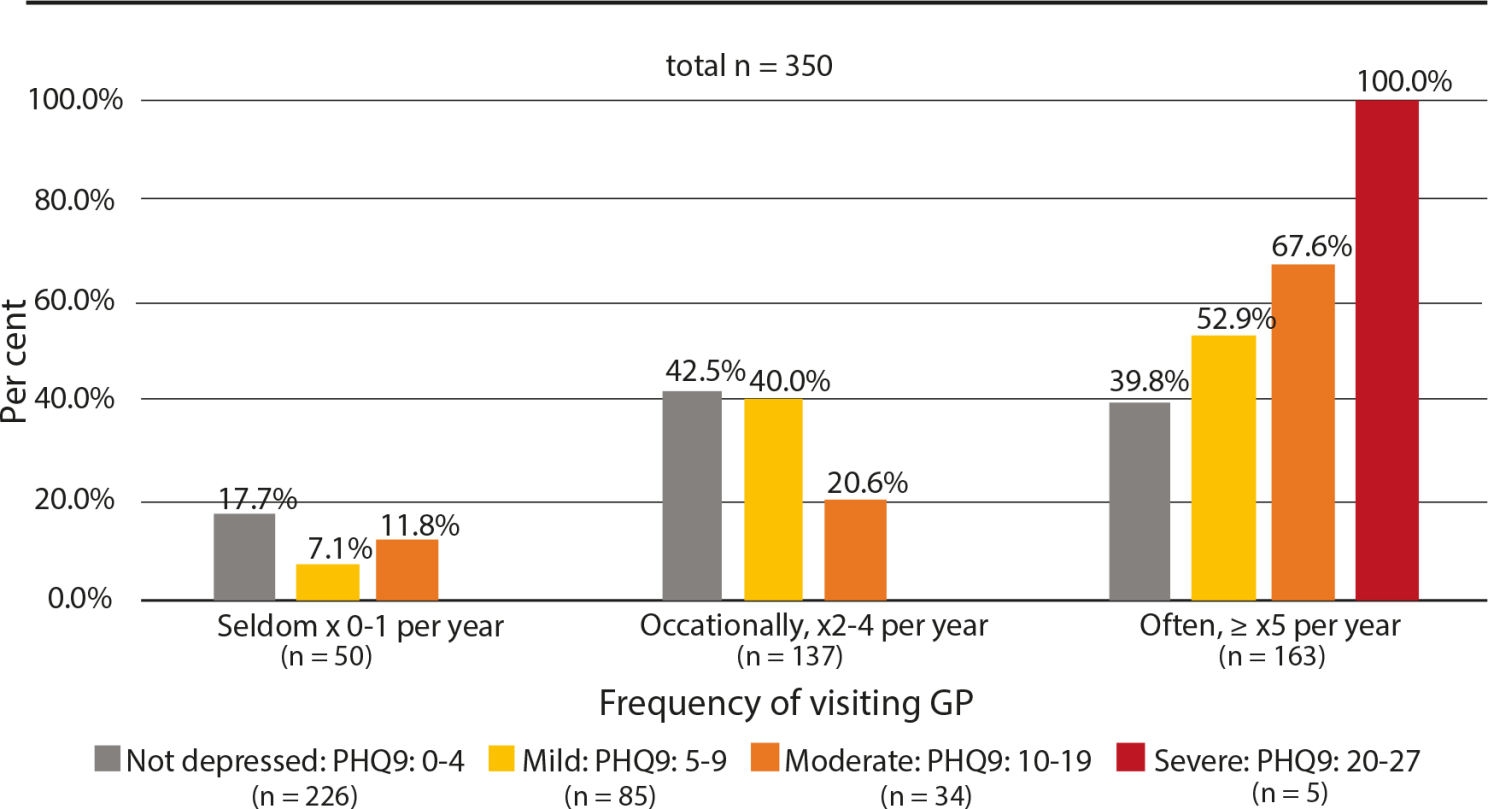
Percentage frequency of consultations, grouped by Patient Health Questionnaire-9 level

Among the moderately depressed patients, 67.6% visited their GP ≥5 times a year (*Figure 3*).

### Additional findings

Table 1 shows various occurrences of depression within different subgroups of the patients. Some of them have very high frequencies of depression, such as patients with foreign native language (28.6%), those who smoke (30.2%), users of anxiolytics (35.2%), users of home-based nursing service (36.4%), those with high subjective health complaints (SHC) ≥20 (23.2%), and those with probable general anxiety disorder GAD-7 ≥10 (72.5%).

While 5.3% of the men and 11.9% of the women in this study addressed mental health issues at the consultation, this sex difference disappeared among those with moderate depressive symptoms. In this group, 32.1% of the women and 30.3% of the men brought this subject into the consultation (*Supplementary Table S1*).

In this sample, there were no sex differences related to the GPs' knowledge of the patient’s depression (*Supplementary Table S1*).

The highest proportion of moderately depressed older people who go to the GP most frequently (≥5 times a year) was predominately women at 79.2%. The proportion of moderately depressed men who go that frequently was only 40.0%, and differs little from men in general (38.0%) (*Supplementary Table 1,*).

All five patients (100%) scoring severe depression (PHQ-9 >19) were known to have a depression by the GP (*Supplementary Table 2, Figure 2),* and they also visited their GP ≥5 times a year (*Supplementary Table 1, Figure 3*).

## Discussion

### Summary

In this study, it was found that 11.4% of the patients aged ≥65 years, in a representative sample of Norwegian general practice, showed moderate or severe depressive symptoms. Only 9.2% of the patients in this study addressed mental health issues at the consultation, while 31.6% of those with moderate depressive symptoms talked about this during the consultation. The GPs were familiar with 57.9% of the patients having moderately depressive symptoms regardless of whether it was brought into the consultation or not. In addition, they also correctly suspected 10.5% more of them to be depressed. Still, 31.6% of the patients with moderate depressive symptoms were unrecognised by their GP. Among those with moderate depressive symptoms, 67.6% visited their GP as frequently as ≥5 times a year.

This study has shown that depression is highly prevalent among older people in Norwegian general practice, but less than one-third of the moderately depressed older patients tended to speak of these symptoms at their consultation. The GPs were familiar with, or suspected, just over two in three of their patients with depression, but still almost one-third remained unrecognised. These patients were frequent visitors at the general practice, but other health issues than depression seemed to be in focus.

### Strengths and limitations

The multi-site nature of the study is a strength but it could be affected by selection bias of GPs who might have been more interested than average in the current topic: older people with depression. The similar findings in the two samples suggest that there was no selection bias (*Table 2*).

The study intentionally used a design to let the GP hand out forms to all the older patients at the end of the consultations in order to remind the GP to fill in the short note of the consultation. It turned out that the GPs often forgot, or did not have time to inform the patient about the study, and the inclusion of patients may therefore have been lower than intended.

In addition, there were a number of situations where medical and practical reasons made it inappropriate to distribute a research form to the patient. Some forms were then sent home, and not returned. Despite the fact that there is no data on non-responders, non-consenters, or GPs' forgotten patients, it is believed that the risk for selection bias is low due to the fact that many of the GPs who recruited few patients did not recruit any patients with depression, only those who were not depressed.

Another issue was that the data collection came during a period of the COVID-19 pandemic, which put GPs under more pressure than usual, and many older patients were reluctant to visit the doctor’s office owing to infection concerns. The findings regarding prevalence must therefore be interpreted with caution.

Selection bias could also potentially have occured in sample 2 where the secretaries invited the patients to participate in the study. On the other hand, one could assume that it would be easier for the patients to decline participation with the secretary than the GP. Altogether, the similarity of the two samples indicates a validity of the findings (*Table 2*).

The number of included patients in the study is limited, which to some extent limits the reliability of the findings. However, it is believed that the major findings presented in this study are reliable as these are not as dependent on the prevalence found, and therefore not affected by the same selection bias that could affect the prevalence.

The fact that there was such a large proportion of patients with depression who were undetected by the GP suggests GPs did not have a selection bias to include more of the patients with depression. And if there had been a selection, the numbers in prevalence would most likely to have been even greater, and the findings of the unrecognised depressions would be even smaller.

### Comparison with existing literature

The prevalence of moderate depression found in this study is somewhat greater than previous studies.^8,9^ Compared with results from the general adult population,^18^ the study found that older patients speak much less of mental health problems. The findings in this study are in line with previous studies regarding older patients’ reluctance to address their mental health issues at the encounter with their GP.^5,23,24,39^

Compared with other studies on patients in the general adult population,^28,29^ the GPs in the present study recognised a greater proportion of older people with depression, which may be owing to differences between countries, culture, practice styles, and age groups. The authors have found no similar studies focusing specifically on older people in general practice. Previous studies have also reported that older patients seem to focus on somatic issues rather than mental health complaints, which leaves the GP unaware of the patient’s mental health issues.^19^

The finding of the patients with depressive symptoms to be among the most frequent GP visitors is also in accordance with previous results.^40,41^

### Implications for research and practice

This study has found that many older patients with moderate depression do not address this at the consultation with their GP. The associations of frequent visitors, unspecific health complaints, and depression among older people should be a catalyst to encourage the GP to discuss mental health issues, which are also known to impact on other chronic conditions.^2^ There is a need for more research that can explore better methods for both recognising and treating older people with depression.

## Data

The dataset relied on in this article is avaliable from the corresponding author on reasonable request.
